# Non‐invasive Imaging Modalities for Breslow Thickness Estimation in Melanoma: A Scoping Review

**DOI:** 10.1111/srt.70392

**Published:** 2026-10-14

**Authors:** Biagio Scotti, Martina Lambertini, Leonardo Veneziano, Federico Venturi, Elisabetta Magnaterra, Carlotta Baraldi, Emi Dika

**Affiliations:** ^1^ Department of Medical and Surgical Sciences *Alma Mater Studiorum* University of Bologna Bologna Italy; ^2^ Oncologic Dermatology Unit IRCCS Azienda Ospedaliero‐Universitaria Di Bologna Bologna Italy

**Keywords:** AI, Breslow, depth, dermoscopy, imaging, melanoma, OCT, RCM, thickness, US

## Abstract

**Background:**

Breslow thickness (BT) is the most important prognostic biomarker in cutaneous melanoma and directly influences surgical management, sentinel lymph node biopsy decisions and survival outcomes. Several non‐invasive imaging modalities have been investigated for preoperative BT estimation; however, their relative performance and clinical applicability remain unclear.

**Objective:**

To evaluate the agreement between non‐invasive imaging‐derived and histopathologically measured BT and to compare the technical characteristics, diagnostic performance, and clinical utility of currently available imaging modalities for preoperative melanoma staging.

**Methods:**

A scoping review was conducted according to Joanna Briggs Institute methodology and reported following PRISMA‐ScR guidelines. MEDLINE, Scopus, and Web of Science were searched from inception to December 1, 2025. Original studies evaluating non‐invasive imaging techniques for BT estimation in cutaneous melanoma were included. Imaging modalities assessed comprised dermoscopy, reflectance confocal microscopy (RCM), optical coherence tomography (OCT), ultrasound (US), multispectral imaging (MSI), photoacoustic techniques, magnetic resonance imaging (MRI), single‐photon emission computed tomography (SPECT), and artificial intelligence (AI)‐based approaches.

**Results:**

Ninety‐two studies met the inclusion criteria. Dermoscopy provided indirect assessment of invasiveness, while RCM and OCT offered complementary microstructural information but limited direct thickness estimation. US, particularly high‐frequency (≥20 MHz) and ultra‐high‐frequency (≥50 MHz) systems, showed the strongest and most consistent evidence for direct BT assessment, with correlation coefficients often exceeding 0.90 and high performance across clinically relevant thresholds. Nevertheless, performance varied across devices, probe frequencies, acquisition protocols, and operators, with frequent overestimation and reduced accuracy in selected subgroups (in situ and thin melanomas). MSI showed moderate‐to‐good classification performance, while photoacoustic techniques yielded promising results but limited to small cohorts. Evidence for MRI and SPECT was scarce. AI‐based approaches achieved moderate‐to‐good predictive performance but did not consistently outperform experienced clinicians, with generalizability limited by dataset and methodological heterogeneity.

**Conclusions:**

US currently represents the most extensively validated and clinically applicable non‐invasive modality for preoperative BT estimation. A hierarchical imaging approach appears most appropriate, with dermoscopy as the first‐line diagnostic tool, RCM and OCT providing additional lesion characterization, and HFUS enabling complementary depth assessment in lesions strongly suspicious for melanoma. Emerging photoacoustic and AI‐based approaches require further validation, while histopathology remains the reference standard for definitive diagnosis and BT measurement.

## 1 Introduction

Breslow thickness (BT) is the most robust and clinically validated prognostic biomarker for cutaneous melanoma, directly influencing survival outcomes and guiding management decisions [1]. Population‐based data show 30‐year melanoma‐specific mortality rates rise from 7.1% for T1 (≤1 mm) to 44.3% for T4 (>4 mm) lesions, demonstrating the critical impact of thickness on prognosis [2, 3]. Moreover, BT provides prognostic value in gene expression profiling (GEP), supporting risk stratification for recurrence in patients with early‐stage disease who did not undergo sentinel lymph node biopsy (SLNB) [4, 5, 6].

In recent years, multiple non‐invasive imaging modalities have been investigated for in vivo assessment of melanocytic lesions, including efforts to estimate BT to support accurate preoperative staging and optimize surgical margin planning. In clinical practice, BT is determined histologically from 2D tissue sections, measuring the maximum depth of melanoma invasion from the granular layer of the epidermis [1, 7].

Conversely, the available literature on non‐invasive imaging techniques for BT estimation remains limited, particularly with regard to direct comparisons between different modalities, as most studies have evaluated individual performances against histopathological measurements alone. Moreover, the optical and hybrid imaging modalities show some limitations, as reflectance confocal microscopy (RCM) only penetrates ∼200 µm, line‐field confocal optical coherence tomography (LC‐OCT) reaches ∼400 µm lacking cellular‐level contrast, high‐frequency ultrasound (HFUS) (≥20 MHz) provides millimetric imaging depth without melanocyte‐specific contrast, and conventional photoacoustic imaging, although highly sensitive to melanin, remains resolution‐limited (>100 µm at ∼25 MHz) [8, 9, 10, 11]. As a result, there is a need for an updated review of the current evidence to map available imaging modalities and inform clinical practice. By integrating findings from clinical studies, we aim to (1) assess the degree of agreement between non‐invasive imaging‐derived and histopathologically measured BT; and (2) compare the technical characteristics, diagnostic performance, and clinical applicability of currently available imaging modalities for preoperative BT estimation.

## 2 Methods

This scoping review was conducted in accordance with the methodological guidance of the Joanna Briggs Institute (JBI) [12]. The Preferred Reporting Items for Systematic Reviews and Meta‐Analyses extension for Scoping Reviews (PRISMA‐ScR) checklist [13] was used to inform and structure the reporting of this review. The review protocol was registered on the Open Science Framework (OSF) Registries during the review process (https://osf.io/7zvtm/overview; accessed on 15 March 2026). Consistent with scoping review methodology, no critical appraisal or risk‐of‐bias assessment of the included studies was performed [12].

### 2.1 Eligibility Criteria

Only articles written in English, German and Italian were considered eligible. To be included in the final analysis, studies had to be published as full‐text original research articles in international, peer‐reviewed journals.

The population, concept, and context (PCC) framework was used to formulate the search question. All studies meeting the following criteria were included in the final review: studies involving patients with cutaneous melanoma (P), evaluated using non‐invasive imaging modalities—such as dermoscopy, RCM, optical coherence tomography (OCT), ultrasound (US), multispectral imaging (MSI), photoacoustic imaging (PAI), magnetic resonance imaging (MRI), and single‐photon emission computed tomography (SPECT)—for the estimation of Breslow thickness (C), within dermatological/clinical diagnostic settings (C).  Studies investigating artificial intelligence or machine‐learning approaches applied to these imaging modalities were also included.

### 2.2 Search Strategy

MedLine (PubMed), Scopus and Web of Science (WOS) databases were searched from inception until 1 December 2025. Full search strategies are detailed in Supplementary Material S1. The search was designed and performed by one author (B.S.) under supervision of a senior investigator (E.D.). No date restriction was applied. Relevant keywords were used for free‐hand searches, and the reference lists of included articles were screened to identify additional sources.

### 2.3 Study Selection and Data Extraction

After removal of duplicate records, two reviewers (B.S. and M.L.) independently screened titles and abstracts for the first‐step evaluation. Subsequently, the same reviewers independently examined the full‐text articles to assess their suitability for inclusion. Preclinical studies relying on cell lines or physical tissue phantoms were excluded. Disagreements were resolved by consultation with a third senior investigator (E.D.), who facilitated consensus. A detailed flowchart of the study selection process is reported in Figure 1.

**FIGURE 1:**
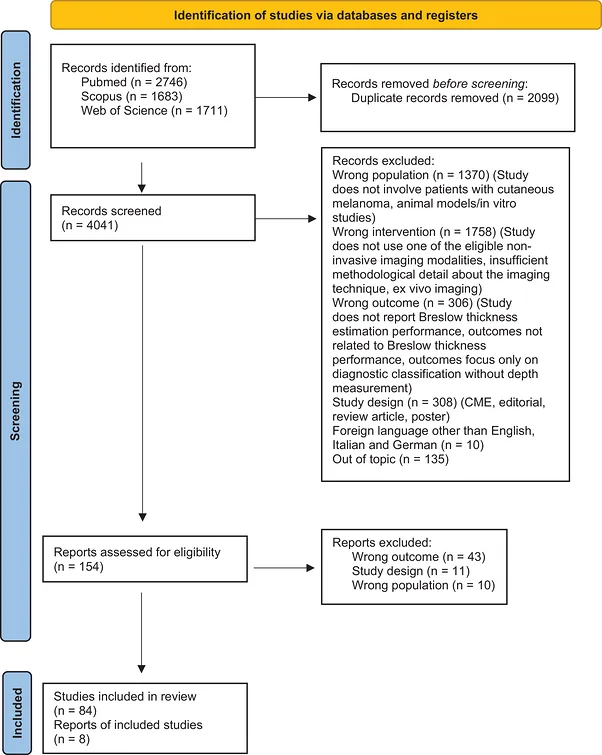
PRISMA diagram.

### 2.4 Synthesis of Results

A narrative synthesis of the extracted data was performed, with findings organized into eight sections according to the non‐invasive imaging modalities evaluated, including a section on artificial intelligence and machine‐learning approaches applied to imaging data.

#### 2.4.1 Dermoscopy

- This section synthesizes evidence on dermoscopic features and scoring approaches used to predict melanoma invasiveness and approximate BT before excision.
- Key outcomes include a consistent association of blue‐white veil, shiny white lines, atypical vessels, milky‐red areas, ulceration, and blue‐gray/‐black pigmentation with thicker melanoma, although precision for exact thickness estimation remains limited.

#### 2.4.2 RCM and OCT

- This section reports studies evaluating RCM and OCT for identifying structural features associated with melanoma thickness.
- Key findings include the association of thicker lesions with cellular and architectural disorganization (non‐edged papillae, pagetoid cells, and epidermal disarray) on RCM, while OCT showed limited reproducibility and a tendency to underestimate thickness.

#### 2.4.3 US

- This section synthesizes the evidence on standard, high‐frequency (≥20 MHz), and ultra‐high‐frequency (≥50 MHz) US for preoperative estimation of BT.
- Key outcomes include consistently strong correlation with histopathology, good performance for clinically relevant thresholds (BT of 1.0 mm), and reduced accuracy in very thin or in situ melanomas with frequent overestimation.

#### 2.4.4 MSI

- This section synthesizes data on MSI for stratifying melanoma by thickness.
- Key outcomes include moderate‐to‐good sensitivity and specificity for thickness classification, suggesting MSI may capture depth‐related optical information beyond conventional dermoscopy.

#### 2.4.5 PAI

- This section synthesizes emerging evidence on PAI techniques for non‐invasive estimation of melanoma depth.
- Key outcomes include promising early accuracy and strong correlation with histopathology in small studies, although more recent reports suggest possible overestimation and less consistent performance.

#### 2.4.6 MRI

- This section synthesizes the limited evidence on MRI‐based assessment of melanoma thickness in selected cases.
- Key outcomes include technical feasibility and moderate‐to‐good agreement with histopathology, but the available evidence is too sparse for routine clinical application.

#### 2.4.7 SPECT

- This section synthesizes exploratory data on SPECT as a functional imaging approach potentially related to melanoma thickness.
- Key outcomes include a positive correlation between tracer uptake and BT, but diagnostic performance was modest and clinical utility for thickness estimation remains limited.

#### 2.4.8 Artificial Intelligence (AI)/Machine Learning (ML)

- This section synthesizes studies applying ML and deep learning models to dermoscopic or multispectral images for thickness prediction.
- Key outcomes include moderate‐to‐good classification performance in binary and multiclass tasks, but inconsistent generalizability and no clear superiority over expert readers in larger studies.

## 3 Results

The search strategy identified 2,746 records in PubMed, 1,683 in Scopus, and 1,711 in WOS. After duplicates’ removal, 4,041 studies proceeded to review. Of these, 3,887 were excluded following screening of titles and abstracts, and full text evaluation was performed on 154 articles. A total of 92 articles were deemed eligible for inclusion in qualitative synthesis (Figure 1). Tables 1 and 2 summarizes the general characteristics and principal findings of the studies included in the qualitative analysis.

**TABLE 1:** Characteristics and main findings of studies included in qualitative synthesis.

Section	Author, year	Country	Type of study	No. of patients	Intervention	Reference standard	Outcome measures	Main findings
Dermoscopy	Reiter et al., 2021	USA	Cross‐sectional study	160 NAM (86 in situ, 74 invasive), 218 DNM (109 in situ, 109 invasive)	Dermoscopy	Breslow thickness	Histopathological and dermoscopic attributes of NAM vs. DNM	Mean BT was greater in lesions with streaks (1.1 mm vs. 0.59 mm), blotches (1.14 mm vs. 0.54 mm), and shiny white structures (1.3 mm vs. 0.54 mm) among invasive NAM/DNM
Dermoscopy	Polesie et al., 2021	Sweden	Retrospective single‐center study	184 MM (110 invasive, 74 in situ)	Dermoscopy	Breslow thickness	SE, SP, concordance (Fleiss’ and Cohen's κ) for prediction of invasiveness	SE and SP for predicting invasiveness ranged from 63.6% to 91.8% and from 32.4% to 82.4%, respectively, across 7 observers. Moderate agreement was observed for melanoma thickness classification (<1.0 vs. ≥1.0 mm; κ = 0.52, 95% CI 0.45–0.58). All invasive melanomas misclassified as in situ were < 1.0 mm. All melanomas >1.0 mm (*n* = 32, 100%) were correctly predicted as invasive by all observers.
Dermoscopy	Ávila et al., 2024	Brazil	Cross‐sectional study	43 MM (21 in situ, 16 thin invasive with BT < 1.0 mm, 6 thick invasive with BT ≥ 1.0 mm)	Dermoscopy	Breslow thickness	Histopathological and dermoscopic attributes of invasive vs. in situ MM, and thick vs. thin invasive MM	Invasive vs. in situ MM: atypical vessels, white scar areas, blue‐white veil, and shiny white structures (all *p* < 0.05). Thick vs. thin invasive MM: radial streaming and blue‐white veil (*p* < 0.05).
Dermoscopy	Togawa et al., 2025	Japan	Cross‐sectional study	94 acral MM (36 in situ, 16 with 0<BT≤1.0 mm, 27 with 1.0<BT≤4.0 mm, 15 with BT>4.0 mm)	Dermoscopy	Breslow thickness	Multivariate analysis: in situ MM vs. MM with BT>4.0 mm	Parallel ridge pattern (OR 23.6; p<0.05), irregular dots (OR 0.11; p = 0.03), blue‐gray clods (OR 0.04; p<0.05), blood crusts (OR 0.02; p<0.05), shiny white lines (OR 0.04; p = 0.01), and blood vessels (OR 0.08; p = 0.02) were associated with thickness category.
Dermoscopy	Mun et al., 2018	Korea	Retrospective multicenter study	75 acral MM (25 in situ, 50 invasive)	Dermoscopy	Breslow thickness	Multivariate analysis: MM with BT>2.0 mm vs. ≤2.0 mm	Red color (OR 16.5; *p* < 0.001), blue color (OR 7.09; *p* = 0.01), white color (OR 5.05; *p* = 0.03), atypical vascular pattern (OR 34.6; *p* < 0.001), blue‐white veil (OR 9.6; *p* = 0.01), and ulceration (OR 5.08; *p* = 0.03) were associated with BT >2.0 mm.
Dermoscopy	Henrik et al., 2001^*^	Denmark	Retrospective single‐center study	65 MM (49 with BT<1.0 mm, 16 with BT>1.0 mm)	Dermoscopy	Breslow thickness	SE, SP, positive/negative likelihood ratio, Fisher's exact test, pre‐/post‐test probability for MM with BT>1.0 mm vs. <1.0 mm	Light‐brown coloration (OR 0.50; *p* < 0.001), pigment network (OR 0.27; *p* < 0.001), irregularity/heterogeneity of the network (OR 0.55; *p* = 0.05), whitish veil (OR 0.28; *p* = 0.06), and pseudopods (OR 0.28; *p* = 0.10) were reported. Gray‐blue areas, atypical vascular patterns, and white scar‐like areas were strong predictors of thick MM (combined presence yielded post‐test probabilities >90%)
Dermoscopy	Seidenari et al., 2010	Italy	Retrospective single‐center study	85 in situ MM, 85 invasive MM, and 85 dermoscopically equivocal lesions histologically diagnosed as nevi	Dermoscopy	Breslow thickness	Multivariate analysis: invasive vs. in situ MM	Regression features (blue areas and/or peppering, blue‐whitish veil, and/or white areas) were more frequent in invasive MM (93% vs. 77%), particularly structureless regression (89% vs. 64%). A reticular pattern of blue regression was a reliable predictor of MM in situ (60% vs. 20%).
Dermoscopy	Podolec et al., 2020	Poland	Retrospective single‐center study	81 MM (22 in situ, 59 invasive)	Dermoscopy	Breslow thickness	SE/SP, Chi‐square test: invasive vs. in situ MM; BT>1.0 mm vs. ≤1.0 mm	Invasive vs. in situ MM: multicomponent pattern (70% vs. 37%, *p* = 0.007; SE 83.7%, SP 43.8%) and pseudopods (31% vs. 9%, *p* = 0.04; SE 90.0%, SP 32.8%). BT >1.0 mm vs. ≤1.0 mm: white regression structures (100% vs. 77%, *p* = 0.04; SE 22.7%, SP 100.0%), atypical blood vessels (60.0% vs. 42.4%; *p* = 0.21), and nodules (60.0% vs. 21%, p = 0.003; SE 39.1%, SP 89.7%).
Dermoscopy	Rubegni et al., 2009	Italy	Retrospective single‐center study	141 MM (108 with BT<1.0 mm, 33 with BT>1.0 mm)	Dermoscopy	Breslow thickness	Multivariate analysis: MM with BT>1.0 mm vs. <1.0 mm	Bluer color (*p* = 0.04) and more random disposition of dermoscopic elements (*p* = 0.04) were associated with thicker melanomas. Overall Acc was 86.5%; 97/108 thin melanomas were correctly classified (SP 89.8%) and 25/33 thick melanomas were correctly classified (SE 75.7%).
Dermoscopy	Verzi et al., 2018	USA	Retrospective case‐control study	1507 melanocytic neoplasms, including 144 MM	Dermoscopy	Breslow thickness	SE, SP, PPV, NPV, Fisher's exact test: MM vs. benign melanocytic neoplasms	Shiny white streaks correlated with greater Breslow depth among melanomas (*p* = 0.007). For MM diagnosis, shiny white streaks showed SE 22% and SP 98%; SP increased to 99.5% when streaks were diffuse across the lesion. PPV and NPV were 58% and 92%, respectively.
Dermoscopy	Carli et al., 2000	Italy	Retrospective single‐center study	84 MM (17 in situ, 67 invasive)	TDS calculated using the ABCD rule of dermoscopy	Breslow thickness	SE, SP, Acc, AUC, ROC analysis. Diagnostic performance of TDS cut‐offs for predicting BT>0.75 mm and BT>1.0 mm;	TDS increased with melanoma thickness from in situ to intermediate lesions (0.75‐1.50 mm). A TDS cut‐off >6.80 identified MM >0.75 mm with 80% SE, 84% SP, 82% Acc, and AUC 0.897. Performance was lower for BT >1.0 mm (AUC 0.810).
Dermoscopy	Stante et al., 2001	Italy	Retrospective single‐center study	84 MM (17 in situ, 67 invasive)	TDS calculated using the ABCD rule of dermoscopy	Breslow thickness	Pearson's chi‐square test; optimized TDS cut‐off (6.80) to predict BT>0.75 mm	Across thickness classes, irregular pigment network (88% in situ; 78% invasive ≤0.75 mm; 33% invasive >1.50 mm; *p* < 0.05, trend *p* = 0.001), radial streaming (6% to 44%; *p* = 0.017), atypical vascular pattern (18% to 56%; *p* = 0.03), and gray‐blue areas (18% to 100%; *p* < 0.001) increased with thickness. TDS >6.80 for detecting melanomas with BT >0.75 mm showed SE 74%, SP 86%, PPV 79%, and NPV 82%.
Dermoscopy	Rodríguez‐Lomba et al., 2021	Spain	Retrospective single‐center study	245 MM (52 in situ, 98 with BT<0.8 mm, 95 with BT ≥0.8 mm)	Dermoscopy	Breslow thickness	SE/SP, PPV, NPV, Pearson's chi‐square test and Fisher's exact test: BT≥0.8 mm vs. <0.8 mm vs. in situ	Red‐pink, blue‐gray, and white color; blue‐white veil; shiny white streaks; irregular vessels; blue‐black pigmentation; milky red areas; pseudolacunae; ulceration; and rainbow pattern were more frequent in thicker lesions (all p<0.01).
Dermoscopy	Pizzichetta et al., 2001	Italy	Retrospective single‐center study	53 MM (19 with BT≤0.75 mm, 34 with BT>0.75 mm)	Dermoscopy	Breslow thickness	Chi‐square test: MM with BT>0.75 mm vs. ≤0.75 mm	Blue‐whitish veil (100% vs. 84%), gray‐blue areas (88% vs. 63%), and linear/dotted vascular pattern (47% vs. 26%) were more frequent in thicker melanomas.
Dermoscopy	Zboraș et al., 2025	Romania	Retrospective single‐center study	52 MM (30 with BT<1.0 mm/stage IA‐IB, 22 in situ/stage 0)	Dermoscopy	Breslow thickness	Concordance (Cohen's κ), Chi‐square or Mann‐Whitney test: MM with BT<1.0 mm vs. in situ	Irregular dots/globules (90% vs. 54%, *p* = 0.008; κ = 0.89), blue‐white veil (77% vs. 41%, *p* = 0.011; κ = 0.96), milky red areas (50% vs. 13%, *p* = 0.008; κ = 0.96), dotted vessels (40% vs. 13%, *p* = 0.04; κ = 0.91), and linear irregular vessels (24% vs. 0%, p = 0.016; κ = 0.79) were associated with invasive thin melanoma compared with in situ disease.
Dermoscopy	Silva et al., 2013	Brazil	Retrospective single‐center study	41 MM (17 in situ, 24 invasive)	Dermoscopy	Breslow thickness	Chi‐square or Fisher's exact test: invasive MM (mean BT 0.47 ± 0.2 mm) vs. in situ MM	Three or more colors (96% vs. 59%; *p* < 0.01), blue‐white veil (38% vs. 0%; *p* = 0.017), milky red area (67% vs. 29%; *p* = 0.02), and atypical network or streaks (67% vs. 35%; *p* = 0.04) were associated with invasive MM.
Dermoscopy	Polesie et al., 2021	Sweden	Retrospective single‐center study	182 MM (101 in situ, 81 invasive)	Dermoscopy	Breslow thickness	Concordance (Fleiss’ κ), multivariate analysis: invasive vs. in situ; BT>1.0 mm vs. ≤1.0 mm	Agreement for invasive vs. in situ classification was moderate (*κ* = 0.52; 95% CI 0.49–0.56), decreasing to fair‐moderate (*κ* = 0.44) for three‐category classification. Invasive vs. in situ: atypical blue‐white structures (OR 15; *p* <0.0001) and polymorphous vessels (OR 3.4; *p* = 0.079). BT>1.0 mm vs. ≤1.0 mm: atypical blue‐white structures (OR 5.7; *p* = 0.017), shiny white lines (OR 5.5; *p* = 0.02), and polymorphous vessels (OR 4.0; *p* = 0.06).
Dermoscopy	Polesie et al., 2022	Sweden	Open, web‐based, international diagnostic study	1456 MM (788 in situ, 474 with BT≤1.0 mm, 194 with BT>1.0 mm)	Dermoscopy and CNNs	Breslow thickness	SE, SP, Acc, ROC analysis, Fisher's exact test (three‐class and binary thickness prediction, comparison between CNNs and reader collective)	Overall Acc was 56.4% (95% CI 55.7%–57.0%), similar between readers with ≥5 years of dermoscopy experience (56.6%) and those with less experience (54.0%). Acc was 85.9% for thin MM and 70.8% for thick MM. Readers performed better on thick vs. thin comparisons than on the 3‐class task. The reader collective (AUC 0.85) outperformed a de novo CNN (AUC 0.80; adjusted *p* = 0.021), but not a pretrained CNN (AUC 0.83; adjusted *p* = 1.0), for differentiating in situ from invasive MM.
Dermoscopy	Gillstedt et al., 2022	Sweden	Retrospective single‐center study	1578 MM (850 in situ, 728 invasive)	CNN combining clinical and dermoscopic images	Breslow thickness	Univariate analysis, concordance (Fleiss’ κ), ROC analysis; comparison of CNN vs. six dermatologists; features associated with in situ vs. invasive MM	The combined dermatologists’ AUC was 0.80 (95% CI 0.77‐0.86), significantly higher than the CNN model (0.73; 95% CI 0.67‐0.78; p = 0.001); three dermatologists significantly outperformed the CNN. Shiny white lines (SP 91.7%), atypical blue‐white structures (SP 97.5%), and polymorphous vessels (SP 97.4%) were associated with invasive and ≥1.0 mm MM; interobserver agreement was moderate (κ = 0.58).
Dermoscopy	Szijártó et al., 2023	Hungary	Retrospective single‐center study	247 MM (Derm7pt dataset: 64 in situ, 183 invasive)	CNN (EfficientNet) using dermoscopic images	Breslow thickness	SE, SP, ROC analysis, three‐class model: thin, intermediate, thick	The model (Acc 74.8%) outperformed prior approaches (reported Acc 68.0% and 64.4%‐68.4%) despite a small training set; performance was projected to improve with larger datasets.
Dermoscopy	Jaworek‐Korjakowska et al., 2019	Poland	Retrospective multicenter study	146 MM (reported subgroups: 58 in situ, 54 with BT<0.76 mm, 46 with 0.76≤BT<1.5 mm, 55 with BT>1.5 mm)^**^	CNN‐based machine learning using a pretrained VGG‐19 model with customized classifier on dermoscopic images	Breslow thickness	TPR, TNR, Acc; three‐class thickness prediction	Thin class: TPR 84.5%, TNR 90.9%, Acc 86.9%. Intermediate class: TPR 78.3%, TNR 87.3%, Acc 85.5%. Thick class: TPR 78.6%, TNR 92.8%, Acc 89.3%. Average: TPR 80.5%, TNR 90.3%, Acc 87.2%.
Dermoscopy	Saez et al., 2016	Spain	Retrospective single‐center study	250 MM (167 with BT<0.76 mm, including 64 in situ; 54 with 0.76<BT<1.5 mm; 29 with BT>1.5 mm)	Machine learning methods for binary and multiclass thickness classification	Breslow thickness	SE, SP, ROC analysis, logistic regression with artificial neural networks (LIPU)	LIPU achieved Acc of 77.6% for binary classification and outperformed other tested algorithms (LR, PUNN, KDA, SVC). For multiclass classification, LIPU Acc was 68.4%.
Dermoscopy	Chu et al., 2022	Korea	Retrospective multicenter study	631 MM (acral lentiginous melanomas only)	Deep learning algorithms from dermoscopic images	Breslow thickness	AUROC analysis; SE, SP; classification: in situ vs. invasive, BT≤0.8 vs. >0.8 mm, BT≤2.0 vs. >2.0 mm	AUROC values for the whole dataset were 0.907 (95% CI 0.888‐0.926) for in situ vs. invasive, 0.793 (95% CI 0.748‐0.838) for BT≤0.8 vs. >0.8 mm, and 0.965 (95% CI 0.953‐0.978) for BT≤2.0 vs. >2.0 mm.
Dermoscopy	Hernández‐Rodríguez et al., 2023	Spain	Retrospective multicenter study	1,315 MM	Deep transfer learning CNN models applied to dermoscopic images	Breslow thickness	ROC analysis, SE/SP, PPV/NPV, balanced Acc	EfficientNetB6 achieved Acc of 61% for MIS vs. invasive MM and 75% for predicting BT<0.8 vs. ≥0.8 mm.
Dermoscopy	Lee et al., 2025	Korea	Retrospective multicenter study	46 MM (LM/LMM only; 32 with BT≤1.0 mm, 14 with BT>1.0 mm)	Dermoscopy	Breslow thickness	SE, SP, ROC analysis, multivariate analysis and predictive risk score for LMM with BT>1 mm	Blue‐white veil (OR 42.895; 95% CI 1.878‐979.565), red rhomboids (OR 13.666; 95% CI 1.070‐174.552), and linear vessels (OR 18.823; 95% CI 1.357‐261.107) were associated with BT>1.0 mm. AUC was 0.964.
Dermoscopy	Argenziano et al., 1999	Italy	Retrospective single‐center study	122 MM (72 with BT<0.76 mm, 31 with 0.76<BT<1.5 mm, 19 with BT>1.5 mm)	Dermoscopy	Breslow thickness	Univariate analysis (chi‐square, Fisher's exact test, Spearman correlation), PPV, Acc; BT<0.76 mm vs. >0.75 mm	PPV was 93% and Acc 67% for MM with BT<0.76 mm; PPV was 69% and Acc 65% for MM with BT>0.75 mm. Palpation predicted thickness category correctly in 61% of cases; combined with dermoscopy, performance improved for MM>0.75 mm (PPV 89%, accuracy 75%) and for MM<0.76 mm (Acc 83%, PPV 88%).
Dermoscopy	Ungureanu et al., 2013	Romania	Case Series	24 MM (11 in situ, 13 with BT <1.0 mm)	Dermoscopy	Breslow thickness	Prevalence of dermoscopic features according to BT	Frequent features were irregular black dots/globules (87.5%), small gray‐blue areas (79.2%), pseudopods (70.8%), and irregular network (62.5%).
Dermoscopy	Silva et al., 2022	Portugal	Retrospective single‐center study	119 MM (59 with BT ≤1.0 mm, 60 with BT >1.0 mm)	Dermoscopy	Breslow thickness	Prevalence of dermoscopic features according to BT	Atypical vascular pattern (65.0%; p<0.001) was associated with BT>1.0 mm. Atypical pigment network/pseudo‐network (86.4%; p<0.001) was associated with BT≤1.0 mm.
Dermoscopy	Satheesha et al., 2017	India	Database study	ATLAS and PH2 datasets	Computerized dermoscopy system proposing 3D skin reconstruction from 2D dermoscopic images	Breslow thickness	Relative depth estimation vs. BT in MM (in situ, 0.5 mm, 0.9 mm)	Relative depth estimates were 0.0918 vs. 0, 0.1388 vs. 0.5, and 0.2437 vs. 0.9 mm. The proposed technique showed a reported difference of 75.58%.
Dermoscopy	Scalise et al., 2009	Italy	Case series	14 pigmented lesions (2 MM)	Multispectral dermoscopy (700/940/950 nm LED illuminator)	Breslow thickness	Relative depth estimation vs. BT in MM	Lesion contrast measured at a single wavelength correlated significantly with histopathology‐derived lesion depth, with best performance at 940–950 nm. The model slightly overestimated maximum MM depth (0.43±0.26 mm).
Dermoscopy	Janssen et al., 2022	Belgium	Retrospective single‐center study	110 MM	Multispectral dermoscopy	Breslow thickness	SE/SP, concordance (Fleiss’ κ): MM with vs. without deep pigment	Mean BT was 1.04 mm. Melanomas with deep pigment on multispectral imaging had greater BT than those without (1.19 vs. 0.68 mm; p = 0.025). Interobserver agreement was good (κ = 0.71; 95% CI 0.651‐0.769).
Dermoscopy	Barragán‐Estudillo et al., 2022	Spain	Retrospective single‐center study	92 MM (28 in situ, 64 invasive; mean BT 1.43±1.6 mm)	Dermoscopy and RCM	Breslow thickness	Chi‐square/Fisher's exact test, Student's t‐test, ANOVA, Pearson's/Spearman's correlation, linear regression: lower vs. higher BT	Lower BT was associated with pigmented network (p = 0.002). Higher BT was associated with visible vessels (p = 0.001) and diffuse inflammatory infiltrates in the superficial dermis (p = 0.04), with a mean BT increase of 0.6 mm.
Dermoscopy	Negrutiu et al., 2024	Romania	Retrospective single‐center study	40 MM (9 in situ)	Dermoscopy and US (20 MHz)	Breslow thickness	Mann‐Whitney or chi‐square test, Spearman correlation, univariate analysis: correlation of dermoscopic and US features with BT	Dermoscopically, atypical network, irregular globules/dots, prominent skin margins, angulated lines/polygons, dotted and short linear vessels, and negative network correlated with median BT≤0.5 mm. Blue‐white veil, atypical vessels, blue‐black color, and milky red color were associated with median BT≥2.3 mm. Dermoscopic vs. histopathologic regression showed moderate agreement (κ = 0.505; p = 0.001). Histologic BT strongly correlated with US‐measured thickness (r = 0.984; p<0.001), highest in NM (r = 0.991) and strong in SSM (r = 0.927). In MIS, US‐measurable thickness was obtained in 5/9 cases.
Dermoscopy	Dummer et al., 1995	Germany	Retrospective single‐center study	108 MM (46 with BT<0.76 mm, 26 with BT 0.76–1.5 mm, 24 with BT1.5‐4.0 mm, 13 with BT>4.0 mm)	Dermoscopy and US (20 MHz)	Breslow thickness	Correlation of dermoscopic features and US with BT	Black dots, grayish‐blue areas, whitish veil within a less characterizable pigment network were present in at least 62% of pT4 MM. US tended to overestimate tumor thickness, mainly because of lymphocytic infiltrate and dermal nevus components adjacent to MM.
**RCM/OCT**	Kardynal et al., 2019	Poland	Retrospective single‐center study	30 MM (15 with BT≤1.0 mm, 15 with BT>1.0 mm)	RCM	Breslow thickness	χ^2^ tests and binary logistic regression: correlation of RCM features with thin vs. thick MM	Thin MM was associated with edged papillae (26.7% vs. 0%; p = 0.032) and areas with honeycomb/cobblestone pattern (33.3% vs. 6.7%; p = 0.068). Thick MM was associated with roundish cells (100% vs. 40%; p = 0.001), non‐edged papillae (100% vs. 60%; p = 0.006), numerous pagetoid cells (73.3% vs. 33.3%; p = 0.028), numerous atypical cells at the dermal‐epidermal junction (53.3% vs. 20%; p = 0.058), and epidermal disarray (93.3% vs. 66.7%; p = 0.068).
RCM/OCT	Garbarino et al., 2020	Italy	Retrospective single‐center study	30 MM	RCM and OCT	Breslow thickness	Correlation of RCM and OCT features with BT	Melanomas showing both cerebriform nests on RCM and icicle‐shaped structures on OCT had greater mean BT than the overall melanoma group (2.4 mm [0.25‐5.5], SD 1.5 vs. 1.9 mm [0.25‐5.5], SD 1.4; p<0.05).
RCM/OCT	Hinz et al., 2012	Germany	Retrospective single‐center study	26 melanocytic tumors, including 3 MM, 1 LM, 1 cutaneous MM metastasis, and benign lesions^**^	US (20 MHz) and OCT	Breslow thickness	Relative depth estimation with US and OCT vs. BT; Spearman correlation	For the 3 MM: 0.63 mm (US) vs. 0.68 mm (OCT) vs. BT 1.5 mm; 0.54 mm vs. 0.56 mm vs. BT 0.45 mm; 0.78 mm vs. 0.77 mm vs. BT 0.65 mm. Considering the whole cohort, r = 0.390 for US and r = 0.734 for OCT.
RCM/OCT	Meyer et al., 2014	France	Prospective single‐center study	67 MM (median BT 0.5 mm, range 0.2–0.9 mm)	US (25 MHz) and OCT (930 nm)	Breslow thickness	ICC, Bland‐Altman, reliability coefficients: relative depth estimation with US and OCT vs. BT	US was reliable and accurate for tumor thickness assessment, with excellent intra‐rater repeatability (G = 0.99), excellent inter‐rater reproducibility (G = 0.97), minimal mean bias (‐0.01 mm), and good agreement (ICC = 0.807). OCT systematically underestimated thickness, with poor reproducibility, mean bias ‐0.53 mm, and ICC 0.00.
**Ultrasound**	Yu et al., 2022	China	Prospective double‐arm single‐center study	30 MM	US (5–18 MHz)	Breslow thickness	Spearman correlation coefficient; PPV; US‐guided vs. clinically guided incisional biopsy	Preoperative US‐guided incisional biopsy improved pathological Acc by targeting the maximum‐thickness area. In the US‐guided group, the lowest PPV was observed in the >4 mm group (2 MM), at 50.0%.
Ultrasound	Shobatake et al., 2018	Japan	Case series	10 MM (3 in situ, 7 invasive; mean BT 3.97 mm in invasive cases)	US (6‐15 MHz, 18 MHz)	Breslow thickness	Prevalence of US features according to BT	Mean histology‐US difference was 0.3 mm. Thickness was underestimated in 2 cases and overestimated in 5, showing an overall tendency toward overestimation, likely due to inflammatory/fibrotic changes or an associated nevus. US appearance was a hypoechoic homogeneous area with well‐defined lower margins in 70%; 30% of in situ MM were not visualized.
Ultrasound	Reali et al., 1989	Italy	Retrospective single‐center study	58 MM (5 in situ, 53 invasive; BT 0.35‐9.2 mm)	US (7.5 MHz)	Breslow thickness	Linear regression; Pearson's correlation coefficient	Strong correlation between US and BT (r = 0.895; p<0.001).
Ultrasound	Ulrich et al., 1999	Germany	Retrospective single‐center study	249 MM	US (7.5 MHz and 20 MHz)	Breslow thickness	Linear regression; Pearson's correlation coefficient	Correlation was moderate with the 7.5 MHz probe (r = 0.76) and strong with the 20 MHz probe (r = 0.94). In pT3/pT4 tumors there were no significant differences between probes; HFUS (20 MHz) was preferable for pT1/pT2 MM. Melanomas appeared as hypoechoic to echo‐poor masses sharply demarcated from surrounding echogenic cutis. Subtumoral infiltrates or nevus remnants may impair Acc.
Ultrasound	Vilana et al., 2009	Spain	Retrospective single‐center study	54 MM (32 with BT≤1.0 mm, 22 with BT>1.0 mm), 45 evaluated with US	US (10 MHz)	Breslow thickness	Pearson's correlation, concordance, SE, SP, Acc, PPV, NPV	Agreement with histopathology was strong (r = 0.93; concordance 0.99). US discriminated BT≤1.0 mm vs. >1.0 mm with SE 86%, SP 97%, Acc 93%, PPV 95%, and NPV 91%.
Ultrasound	Shafir et al., 1984	Israel	Prospective single‐center study	15 MM (mean BT 1.95±1.47 mm)	US (10 MHz)	Breslow thickness	Linear regression analysis	Strong correlation between US and histologic thickness (r^2^ = 0.92).
Ultrasound	Schwaighofer et al., 1987	Austria	Case series	26 MM	US (10 MHz)	Breslow thickness	Correlation between US and BT	Good correlation; US measurements were generally higher than histometric ones. SSM appeared as a hyperechoic band, whereas NM presented as a hypoechoic to nearly anechoic structure with well‐defined margins.
Ultrasound	Music et al., 2010	Slovenia	Prospective single‐center study	60 MM (24 with BT≤1.0 mm, 36 with BT>1.0 mm)	US (12‐15 MHz)	Breslow thickness	Pearson's correlation, concordance, SE, SP, PPV, NPV	For MM with BT >1.0 mm, SE was 92%, SP 92%, PPV 95%, and NPV 89%. Correlation with histology was good (r = 0.82; p<0.001), increasing to r = 0.90 after excluding two MM >7.5 mm.
Ultrasound	Kučinskienė et al., 2014	Lithuania	Retrospective single‐center study	46 melanocytic tumors (12 MM, 34 nevi)^**^	US (14 MHz)	Breslow thickness / vertical tumor thickness	Chi‐square, Spearman correlation, SE, SP, Acc, PPV, NPV	US/histologic correlation was weak in thin tumors (r = 0.417) and moderate in thick tumors (r = 0.694). In homogeneous tumors (n = 43), US thickness was 1.14 mm vs. histometry 1.72 mm, with moderate correlation (r = 0.567).
Ultrasound	Kaikaris et al., 2011	Lithuania	Prospective single‐center study	100 MM	US (14 MHz)	Breslow thickness	Chi‐square, Fisher's exact test, Pearson's correlation, Bland‐Altman	Correlation between sonometric and histometric thickness was weak in thinner MM (BT 1–2.0 mm; r = 0.283) and strong in thicker MM (BT>2.0 mm; r = 0.869; p<0.0001).
Ultrasound	Fernández Canedo et al., 2013	Spain	Retrospective single‐center study	79 MM (36 in situ, 27 with BT≤1.0 mm, 16 with BT>1.0 mm)	US (15 MHz)	Breslow thickness	Chi‐square, Fisher's exact test, Pearson's correlation, ICC, SE, SP, PPV, NPV	Correlation/agreement was moderate (Pearson r = 0.678; ICC 0.78). For MM >1 mm, SE was 82%, SP 80%, PPV 54%, and NPV 94%.
Ultrasound	Yu et al., 2023	China	Retrospective single‐center study	105 acral MM (mean BT 3.9±2.3 mm)	US (18 MHz)	Breslow thickness	PPV, Spearman correlation, Bland‐Altman	Correlation was strong (r = 0.83; p<0.001). US showed high PPV overall (mean 84.6%), with lowest values in BT 1–2.0 mm lesions (PPV 50%) and in situ lesions (PPV 60%).
Ultrasound	Lassau et al., 1999	France	Prospective single‐center study	27 MM (16 with BT<1.0 mm, 5 with 1.01<BT<4.0 mm, 6 with BT>4.0 mm)	US (20 MHz)	Breslow thickness	Pearson's correlation coefficient	Thickness ranged from 0.30‐8.0 mm by US vs. 0.26‐8.0 mm by histology, with strong correlation (r>0.950).
Ultrasound	Hoffmann et al., 1992	Germany	Retrospective single‐center study	54 MM	US (20 MHz)	Breslow thickness	Relative depth estimation vs. BT	Correlation was strong (r = 0.938). The greatest deviation occurred in MM with BT <0.75 mm.
Ultrasound	Hoffmann et al., 1999	Germany	Prospective multicenter study	264 MM	US (20 MHz)	Breslow thickness	Pearson's correlation coefficient	Excellent correlation was observed between US and BT (r = 0.97), whereas palpation showed lower correlation (r = 0.59).
Ultrasound	Pennasilico et al., 1994	Italy	Prospective single‐center study	29 MM (12 with BT<1.0 mm, 10 with BT 1.0–2.0 mm, 7 with BT>2.0 mm)	US (20 MHz) A‐mode	Breslow thickness	Comparison of US vs. BT	Ultrasound closely approximated BT but tended to overestimate it by about 7–11%, with the discrepancy increasing with tumor thickness.
Ultrasound	Rompel et al., 1993	Germany	Case series	3 MM	US (20 MHz)	Breslow thickness	Descriptive analysis of ultrasonographic patterns	MM typically appeared as homogeneous hypoechoic structures with sharp margins; ultrasound may estimate invasion depth preoperatively.
Ultrasound	Rompel et al., 1997	Germany	Prospective single‐center study	155 MM	US (20 MHz)	Breslow thickness	Spearman's correlation coefficient	Strong correlation was found between US and BT (r = 0.890), with mean sonometric deviation 0.25 mm (SD 0.35). Correlation was better for thick (0.76‐1.50 mm; r = 0.70) than thin (≤0.76 mm; r = 0.46) MM.
Ultrasound	Solivetti et al., 1998	Italy	Prospective single‐center study	78 melanocytic tumors, including 66 MM and 12 nevi^**^	US (20 MHz)	Breslow thickness	Pearson's correlation coefficient	Strong correlation between US and BT (r = 0.930).
Ultrasound	Gassenmaier et al., 1990	Germany	Prospective single‐center study	72 MM	US (20 MHz)	Breslow thickness	Pearson's correlation coefficient	Strong correlation was found (r = 0.97). In two cases, US underestimated histometric thickness.
Ultrasound	Tacke et al., 1995	Germany	Retrospective single‐center study	259 MM	US (20 MHz)	Breslow thickness	PPV, Pearson's correlation, ICC	Correlation/agreement was good (r = 0.880; ICC 0.75), with mean difference 0.39 mm (relative difference 28%). US thickness matched histometry in 75% of cases.
Ultrasound	Pellacani et al., 2003	Italy	Retrospective single‐center study	88 MM (60 with BT≤1.0 mm, 28 with BT>1.0 mm)	US (20 MHz)	Breslow thickness	Chi‐square, Fisher's exact test, SE, SP, Spearman correlation, Bland‐Altman	Correlation was good (r = 0.89; p<0.001). Thin MM were correctly classified in 85% of cases; thick MM in 93–100%.
Ultrasound	Salmhofer et al., 1996	Austria	Retrospective single‐center study	18 MM	US (20 MHz)	Breslow thickness	Pearson's correlation coefficient	Strong correlation was observed (r = 0.93), with mean absolute difference 50.3 µm between US and histologic measurements.
Ultrasound	Partsch et al., 1996	Austria	Prospective single‐center study	71 MM (13 in situ)	US (20 MHz)	Breslow thickness	Bland‐Altman and least‐squares regression	Strong correlation was observed (r = 0.92), but Acc was lower than previously reported.
Ultrasound	Machet et al., 2009	France	Prospective single‐center study	31 MM (mean BT 1.96±2.15 mm)	US (20 MHz)	Breslow thickness	Bland‐Altman, concordance (Cohen's κ)	Strong correlation was reported (r = 0.94); US overestimated thickness in 17 cases and underestimated it in 10.
Ultrasound	Kozárová et al., 2018	Slovakia	Retrospective single‐center study	50 MM (26 with BT≤1.0 mm, 17 with BT 1.01‐2.00 mm, 7 with BT>2.0 mm)	US (20 MHz)	Breslow thickness	Spearman's correlation coefficient	Strong correlation was found (r = 0.92; p<0.001). Mean US thickness was 6.77% higher than mean BT.
Ultrasound	Hoffmann et al., 1989	Germany	Retrospective single‐center study	26 MM	US (20 MHz)	Breslow thickness	Correlation between US and BT	Sonographic thickness correlated well with histometric measurements from paraffin sections despite tissue shrinkage.
Ultrasound	Andrekute et al., 2016	Lithuania	Retrospective single‐center study	52 melanocytic tumors (6 MM, 46 nevi; BT≤1.0 mm)^**^	US (22 MHz): automated and manual measurements	Breslow thickness	Spearman's correlation, SE, SP	Automated US measurements showed strong correlation (r = 0.83; p<0.0001), SE 96.6%, SP 78.3%. Manual US measurements showed weaker correlation (r = 0.68; p<0.0001).
Ultrasound	Linkeviciute et al., 2018	Lithuania	Retrospective single‐center study	54 MM	US (22 MHz)	Breslow thickness	Spearman correlation, Bland‐Altman	Good correlation was found (r = 0.86; p<0.001). Bland‐Altman plots showed mean difference ‐0.07 mm (95% CI ‐0.18 to 0.04 mm).
Ultrasound	Sakalauskienė et al., 2018	Lithuania	Prospective single‐center study	102 melanocytic tumors: 43 MM, 59 nevi^**^	US (22 MHz) and SIAscopy	Breslow thickness	SE/SP, ROC, Pearson's correlation; classification BT ≤1.0 mm vs. >1.0 mm	US: r = 0.73 (p<0.001). SIAscopy showed SE 81.4% and SP 86.4% (AUC 0.90‐0.96), but these results referred to the whole melanocytic lesion cohort rather than melanoma‐only BT estimation.
Ultrasound	Serrone et al., 2002	Italy	Retrospective single‐center study	193 MM (31 in situ, 62 with BT≤0.75 mm, 57 with 0.76<BT<1.5 mm, 35 with 1.51<BT<4.0 mm, 8 with BT>4.0 mm)	US (20 MHz, 13 MHz)	Breslow thickness	Pearson's correlation coefficient	Strong correlation was found (r = 0.95), with absolute difference 0.32±0.03 mm and mean relative difference 27.2% (95% CI 23–31.4%).
Ultrasound	Bessoud et al., 2003	France	Retrospective single‐center study	74 MM (correlation assessed in 65 cases)	US (20 MHz, 13 MHz)	Breslow thickness	Pearson's correlation coefficient	Strong correlation was found (r>0.96; p<0.001).
Ultrasound	Crisan et al., 2013	Romania	Prospective single‐center study	28 MM (8 SSM, 20 NM)	US (20 MHz, 7–14 MHz)	Breslow thickness	Best‐fit correlation model	Correlation coefficients were 99.4% for SSM and 98.4% for NM with the 20 MHz probe.
Ultrasound	Piłat et al., 2020	Poland	Prospective single‐center study	19 MM (mean BT 0.56±0.47 mm)	US (20 MHz, 50 MHz)	Breslow thickness	Spearman's correlation coefficient	Correlation was ρ = 0.759 (p<0.001) with the 20 MHz probe and ρ = 0.734 (p<0.001) with the 50 MHz probe.
Ultrasound	Hayashi et al., 2009	Japan	Prospective single‐center study	74 MM (15 with BT≤1.0 mm, 16 with 1.1<BT<2.0 mm, 19 with 2.01<BT<4.0 mm, 18 with BT>4.0 mm)	US (15 MHz, 30 MHz)	Breslow thickness	PPV, Pearson's correlation coefficient	Correlation was good (r = 0.887), especially for foot melanomas (n = 26; r = 0.945).
Ultrasound	Maj et al., 2015	Poland	Retrospective single‐center study	19 MM (11 in situ, 8 invasive; BT 0.2‐7.0 mm)	US (30 MHz)	Breslow thickness	Relative depth estimation vs. BT	In invasive MM, US thickness was 10.7% higher than BT.
Ultrasound	Varga et al., 2023	Hungary	Prospective single‐center study	101 MM (mean BT 1.61±1.69 mm; range 0.135‐8.12 mm)	OG‐HFUS (20–40 MHz) and MSI	Breslow thickness	Pearson's correlation, SE, SP, PPV, NPV, Cohen's κ, multivariate analysis	For OG‐HFUS, agreement across categories (<1.0 mm, 1–2.0 mm, >2.0 mm) was high (κ = 0.858), with SE 91.8%, SP 96%, PPV 91.8%, NPV 96%. MSI showed lower agreement (κ = 0.44).
Ultrasound	Turnbull et al., 1995	Canada	Case Report	1 MM	Ultrasound backscatter microscope (62 MHz)	Breslow thickness	Relative depth estimation vs. BT	US thickness was 0.37 mm vs. 0.42 mm by histology.
Ultrasound	Oranges et al., 2023	Italy	Retrospective single‐center study	27 MM (1 in situ, 17 with BT<1.0 mm, 6 with 1.0<BT<2.0 mm, 2 with 2.01<BT<4.0 mm, 1 with BT>4.0 mm)	US (70 MHz)	Breslow thickness	Pearson's correlation, coefficient of variation, ICC, Bland‐Altman	Correlation was strong (mean r = 0.98 across two operators), agreement was excellent (CV 6.5%; ICC 0.99), and Bland‐Altman analysis showed no significant bias.
Ultrasound	Reginelli et al., 2020	Italy	Case Series	14 MM (12 in situ)	US (70 MHz)	Breslow thickness	Comparison of US‐estimated depth vs. BT in 7/14 MM	US and BT values were closely matched in the 7 lesions assessed.
Ultrasound	Guitera et al., 2008	Australia	Prospective single‐center study	55 MM (measurements available in 45; median BT 0.4 mm, range 0.1‐0.6 mm; 22 in situ)	US (75 MHz)	Breslow thickness	Pearson's correlation, Lin's concordance correlation coefficient	Median percentage error was 13%; correlation was strong (r = 0.908; p<0.001). No melanomas with BT<1.0 mm were misclassified as >1.0 mm.
Ultrasound	Gambichler et al., 2007	Germany	Prospective single‐center study	13 MM	US (100 MHz, 20 MHz)	Breslow thickness	Pearson's correlation, coefficient of repeatability, Bland‐Altman	Strong correlation was reported for both probes (r = 0.99; p<0.001). Both methods overestimated BT, with larger bias for 100 MHz.
Ultrasound	Botard‐Jid et al., 2015	Romania	Prospective single‐center study	42 MM (5 in situ)	US (40 MHz) and strain elastography (SE)	Breslow thickness	Fisher's exact test, Pearson's correlation, Spearman's correlation	US showed strong correlation with BT (r = 0.959). For SE, a weak inverse association with thickness was reported (ρ = ‐0.3052; p = 0.04).
Ultrasound	Berghe et al., 2020	Romania	Retrospective single‐center study	52 MM	US (40 MHz) and SE	Breslow thickness	Spearman's correlation, ROC, Bland‐Altman; thin vs. intermediate/thick classification	US distinguished thin from thicker melanomas with AUC 0.970 (95% CI 0.927‐1.0). Elastographic strain ratio showed limited Acc (AUC 0.739).
**MSI**	Bozsányi et al., 2021	Hungary	Retrospective single‐center study	100 MM (mean BT 1.77±1.73 mm)	MSI	Breslow thickness	Pearson's correlation, SE, SP, Cohen's κ, ROC; 3‐group classification	Overall SE was 78%, SP 89%, and agreement κ = 0.67. Performance was better than algorithms based only on dermoscopic and clinical images.
MSI	Marchesini et al., 2007	Italy	Retrospective single‐center study	250 MM (median BT 0.55 mm, range 0.12‐2.7 mm)	ANN from MSI images	Breslow thickness	SE/SP, ROC; BT<0.75 mm vs. ≥0.75 mm	Overall SE ranged from 76% to 90% and SP from 74% to 91%.
**Photoacoustic**	Zhou et al., 2017	USA	Prospective single‐center study	10 MM (BT 0.2‐6.0 mm)	PAT (680 nm)	Breslow thickness	Mean absolute error and coefficient of determination	Corrected PAT depth showed MAE 0.18 mm and R^2^ = 0.97.
Photoacoustic	von Knorring et al., 2025	Denmark	Prospective single‐center study	75 MM	PAI	Breslow thickness	Relative depth estimation vs. BT	PAI tended to overestimate tumor thickness compared with histopathology and did not confirm the high Acc reported in earlier work.
Photoacoustic	Breathnach et al., 2018	Ireland	Prospective single‐center study	32 melanocytic tumors (6 MM, 26 nevi)^**^	PAI (256 acoustic transducers; central frequency 40 MHz)	Breslow thickness	Two‐tailed t‐test; correlation coefficient	Strong correlation with BT was reported (r = 0.99; p<0.001).
Photoacoustic	Park et al., 2020	Korea	Prospective single‐center study	6 MM (1 in situ; mean BT 3.38±2.86 mm)	Multispectral PAI (700‐900 nm)	Breslow thickness	MAE	Melanin‐unmixed PAI depth showed good agreement with BT (MAE 0.36 mm).
Photoacoustic	Aguirre et al., 2025	Germany	Prospective single‐center study	7 MM	UWB‐RSOM (650 nm, 10–100 MHz)	Breslow thickness	Linear regression, coefficient of determination	Excellent correlation with BT was reported (R^2^ = 0.886; p = 0.0002).
Photoacoustic	Fedorov Kukk et al., 2025	Germany	Retrospective single‐center study	52 melanocytic tumors (2 MM, 50 nevi)^**^	PAT/US imaging system, OCT, Raman spectroscopy	Breslow thickness	Bland‐Altman, coefficient of determination	PAT/US showed R^2^ = 0.97 and tended to overestimate thickness by 0.1‐0.2 mm. OCT was limited by penetration depth (∼0.5 mm).
**MRI**	Kang et al., 2020	Korea	Retrospective single‐center study	11 MM (mean BT 4.31±2.05 mm)	MRI (1.5 T, 47‐mm microscopy coil)	Breslow thickness	ICC, CCC: T2WI and Gd‐T1WI vs. BT	T2‐weighted imaging correlated better with histopathology than Gd‐T1WI. T2WI: CCC 0.64‐0.78, ICC 0.93‐0.95. Gd‐T1WI: CCC 0.50‐0.61, ICC 0.93‐0.96.
MRI	Nakayama‐Takeda et al., 2013	Japan	Retrospective single‐center study	1 MM with BT 2.06 mm	MRI (1.5 T, 47‐mm or 23‐mm microscopy coil)	Breslow thickness	Relative depth estimation vs. BT	MRI‐measured tumor thickness was 2.07 mm.
**SPECT**	Masiero et al., 2013	Brazil	Prospective single‐center study	20 melanocytic tumors: 14 MM (5 in situ), 6 nevi^**^	SPECT using 99mTc‐sestamibi	Breslow thickness	Spearman's correlation: uptake intensity vs. BT	SE was 64.3% and SP 66.7% for MM detection. A strong positive correlation was found between uptake intensity and BT (rs = 0.74; p = 0.003).

Legenda: ABCD  = asymmetry, border, color, differential structural components; Acc  = accuracy; AUC  = area under the curve; AUROC  = area under the receiver operating characteristic curve; BT  = Breslow thickness; CI  = confidence interval; CNN  = convolutional neural network; CCC  = concordance correlation coefficient; DNM  = de novo melanoma(s); Gd  = gadolinium; HFUS  = high‐frequency ultrasound; ICC  = intraclass correlation coefficient; KDA  = kernel discriminant analysis; LM  = lentigo maligna; LMM  = lentigo maligna melanoma; LR  = logistic regression; MAE  = mean absolute error; MIS  = melanoma in situ; MM  = melanoma(s); MSI  = multispectral imaging; NAM  = nevus‐associated melanoma(s); NM  = nodular melanoma; NPV  = negative predictive value; OCT  = optical coherence tomography; OG‐HFUS  = optically guided high‐frequency ultrasound; PAI  = photoacoustic imaging; PAT  = photoacoustic tomography; PPV  = positive predictive value; PUNN  = product unit neural network; RCM  = reflectance confocal microscopy; ROC  = receiver operating characteristic; RSOM  = raster‐scanning optoacoustic mesoscopy; SE  = sensitivity; SEM  = standard error of the mean; SIAscopy  = spectrophotometric intracutaneous analysis; SP  = specificity; SPECT  = single‐photon emission computed tomography; SSM  = superficial spreading melanoma; SVC  = support vector classifier; T = Tesla; TDS  = total dermoscopy score; UBM  = ultrasound backscatter microscopy; US  = ultrasound; UWB  = ultra‐wide bandwidth; WI  = weighted images; κ  = kappa coefficient.

/ (not specified) ^*^ Multicomponent pattern: ≥3 areas representing different dermoscopic features within a single lesion. ^**^ When statistical results for Breslow thickness in melanoma could not be separated from those of other melanocytic tumors, the entire study population was retained. ^***^ In some RSOM/PAT studies, BT was defined as the distance from the skin surface to the deepest tumor cell.

**TABLE 2:** Technical features and clinical applicability of non‐invasive imaging modalities for Breslow thickness estimation.

Imaging modality	Space resolution	Maximum penetration depth	Direct BT estimation	Clinical maturity	Main strength	Main limitation
Dermoscopy	Low to moderate	Surface	No	High	Widely available; rapid initial assessment	Indirect BT estimation
US/HFUS	Moderate	mm‐cm	Yes	High	Best validated direct BT estimation	Overestimation; operator/device dependence
RCM	Very high (∼1 µm)	∼200 µm	No	Moderate	Cellular‐level resolution	Limited penetration depth
OCT	Moderate (5–15 µm)	∼1–2 mm	Limited	Moderate‐Low	Cross‐sectional structural imaging	Underestimation/limited melanoma evidence
MSI	Moderate	Variable	Indirect	Emerging	Non‐contact quantitative optical information	Limited evidence; indirect estimation
PAT/PAI	High	mm‐cm	Yes	Experimental	Melanin‐sensitive depth assessment	Experimental; limited validation
MRI	Moderate	Deep	Yes	Low	Deep tissue assessment	Cost and limited evidence
SPECT	Low	Whole body	No	Low	Functional whole‐body imaging	Poor applicability to local BT estimation

Supplementary material S1. Search strings used for the three databases.

### 3.1 Dermoscopy

Dermoscopy (dermatoscopy or epiluminescence microscopy) is a noninvasive imaging technique that uses a handheld dermatoscope to magnify skin lesions, allowing assessment of subsurface colors and microstructures invisible to the naked eye [14, 15].

The evidence from dermoscopic studies shows specific morphologic features correlating with melanoma invasiveness and increasing BT [16, 17, 18, 19, 20, 21, 22, 23, 24, 25, 26, 27, 28, 29, 30, 31, 32, 33, 34, 35, 36, 37, 38, 39, 40, 41, 42, 43, 44, 45, 46, 47, 48, 49]. Across multiple cohorts, features reflecting dermal involvement and tumor progression, such as blue‐white veil [18, 20, 21, 22, 28, 30, 31, 32, 34, 40, 48, 49], shiny white structures/lines [16, 18, 19, 25, 28, 29, 31, 32, 34], atypical or polymorphous vessels [18, 20, 21, 23, 27, 32, 34], milky‐red areas [20, 28, 48], ulceration [19, 20] and blue‐gray/blue‐black pigmentation [16, 20, 24, 27, 28, 29, 32, 42, 47, 48, 49] were repeatedly associated with thicker lesions. These findings were consistent across different melanoma subtypes, including acral and lentigo maligna melanoma [19, 20, 38, 40].

Conversely, features such as pigment network and reticular patterns were more frequently associated with melanoma in situ or thin invasive (BT<1.0 mm) melanoma [21, 22, 27, 31, 47, 48].

Despite its reproducibility and widespread clinical utility, dermoscopy provides an indirect estimate of BT and lacks sufficient accuracy for precise quantitative measurement.

Interobserver agreement was only moderate [17, 32, 34, 41, 48], particularly when multiple (≥3) thickness categories of depth were considered, and misclassification occurred mainly among in situ and thin invasive melanomas. Notably, lesions thicker than 1.0 mm were almost always correctly identified as invasive [16, 17, 20, 21, 23, 24, 28, 30] indicating that dermoscopy alone performs better at identifying clearly advanced lesions than at resolving subtle distinctions near clinically relevant thresholds.

Scoring systems such as the total dermoscopy score (TDS) showed reasonable diagnostic performance for identifying melanomas above lower thresholds (e.g., 0.75 mm) [26, 27], but their accuracy decreased for higher thresholds (e.g., 1.0 mm) [26], further supporting the limitation of rule‐based approaches for precise stratification.

### 3.2 RCM and OCT

RCM and OCT are noninvasive imaging techniques that provide in vivo assessment of skin architecture beyond dermoscopy. RCM uses an 830‐nm laser to obtain cellular‐resolution (∼1 µm) en face images of the epidermis and superficial dermis (200µm depth), while OCT uses 1310‐nm light to generate cross‐sectional and 3D images with deeper penetration (up to 2 mm) and a resolution of 3–15 µm [50, 51].

The available evidence on RCM and OCT suggests that these modalities are better suited for microstructural characterization rather than direct thickness measurement [52, 53, 54, 55].

RCM findings showed a clear relationship between increasing BT and architectural disorganization, including non‐edged papillae [52], pagetoid cells [52] and epidermal disarray [52, 53]. In contrast, features such as edged papillae and preserved honeycomb patterns were more common in thin melanomas [52].

OCT, either alone or in combination with RCM, showed inconsistent performance [53, 54, 55]. While certain structural patterns were associated with thicker melanomas (icicle‐shaped structures) [53], prospective data demonstrated that OCT systematically underestimated tumor thickness [54, 55] and had poor reproducibility [53, 54, 55]. This limitation is likely related to its restricted penetration depth and technical variability.

### 3.3 US

Skin US is a noninvasive imaging technique that uses ultrasound waves to evaluate the skin and its appendages. High‐ and ultra‐high‐frequency transducers enable detailed visualization of cutaneous structures by optimizing both resolution and penetration depth [56]. US demonstrated the most robust and consistent performance among all modalities reviewed [57, 58, 59, 60, 61, 62, 63, 64, 65, 66, 67, 68, 69, 70, 71, 72, 73, 74, 75, 76, 77, 78, 79, 80, 81, 82, 83, 84, 85, 86, 87, 88, 89, 90, 91, 92, 93, 94, 95, 96, 97, 98, 99, 100]. This was particularly evident with high‐frequency (≥20 MHz) probes, where correlation coefficients frequently exceeded 0.90 across both retrospective and prospective investigations [69, 70, 71, 75, 76, 79, 80, 81, 82, 87, 88, 91, 95, 97, 98, 99, 100]. Nevertheless, several studies have reported good diagnostic performance with concordance coefficients ranging from 0.82 to 0.92 using probes operating at frequencies below 20 MHz [59, 61, 62, 64, 68]; importantly, US also performed well in clinically relevant threshold‐based classifications [64, 66, 67, 69, 70, 74, 77, 93, 94].

In this sense, several studies reported high sensitivity and specificity for distinguishing melanomas ≤1.0 mm from those >1.0 mm, with accuracy rates over 90% [61, 64, 69, 82, 87, 91]. Furthermore, recent developments including optically guided high‐frequency ultrasound (OG‐HFUS) and very‐high‐frequency probes (≥50 MHz) [95, 98, 99], have shown promising agreement with histopathological measurements, including in thinner lesions [96, 97, 100].

Even so, US was not without limitations and increasing probe frequency did not consistently translate into improved diagnostic performance. A consistent finding across studies was a tendency to overestimate tumor thickness [58, 60, 63, 68, 72, 74, 75, 77, 78, 79, 81, 82, 87, 92, 98] both for ≤20 MHz probes [58, 63, 68, 72, 81, 82], 21–49 MHz probes [92] and ≥50 MHz probes [98]. Although underestimation was less frequently reported, it also occurred [65, 75, 76, 81, 94].

Accuracy was reduced in melanoma in situ which may not be visualized at all [58, 68, 75], in thin (<0.75/1.0 mm) [70, 81, 82] and thinner melanomas (1.0–2.0 mm vs. 2.0–3.0 mm or ≤0.76 vs. 0.76–1.5 mm) [66, 68, 74, 76, 80] using ≤20 MHz probes. The worst performances may also occur exactly in the clinically critical range (∼0.75–1.5 mm) whereas higher predictive values are observed at the extremes of BT (<0.55 mm and >1.7 mm) [77]. Nevertheless, some authors reported a high degree of concordance with ≤20 MHz probes even in melanomas with BT <1.0 mm [81, 82, 89, 91], as well as others demonstrated improved accuracy in this subgroup when US assessment was combined with AI [84].

Overall, when BT was assessed with ≤20 MHz probes, the highest accuracy has been reported for melanomas with a BT ranging from 1.5 mm [60, 61, 62, 65, 70, 72, 74, 87] or 2.0 mm [66, 68, 78, 82] up to 4.0 mm [57, 64, 66].

### 3.4 MSI

MSI is a non‐invasive imaging technique that acquires images at multiple wavelengths across the visible and near‐infrared spectrum (400–970 nm). By exploiting the distinct optical properties of skin chromophores, such as melanin and hemoglobin, MSI provides information on pigmentation, vascularity, and tissue composition [101].

In stratifying melanoma thickness, it showed moderate‐to‐good performance, although the evidence base remains limited compared with dermoscopy and US [102, 103]. Nevertheless, two retrospective studies reported sensitivity and specificity values ranging from approximately 75%–90%, considering shape descriptors and intensity values [102, 103] and categorizing the thickness into three [102] or two [103] classifications. Despite encouraging results, particularly when combined with artificial neural networks (ANN) [103], MSI should presently be considered an adjunctive rather than a standalone diagnostic modality.

### 3.5 Photoacoustic Techniques

Photoacoustic techniques combine optical excitation with ultrasound detection. Short laser pulses are absorbed by endogenous chromophores, particularly melanin and hemoglobin, generating thermoelastic expansion and US waves that can be detected and reconstructed into images [104].

Photoacoustic tomography (PAT) was evaluated by Zhou et al. in 10 melanomas and showed excellent agreement with histopathology, with a mean absolute error of 0.18 mm and a great coefficient of determination (*R* ^2^ = 0.97) [105]. Similarly, Fedorov Kukk et al. reported excellent correlation (*R* ^2^ = 0.97) using a combined PAT/US system, although thickness was consistently overestimated by approximately 0.1–0.2 mm [106].

PAI was also assessed in two prospective studies. Breathnach et al. found an almost perfect correlation between photoacoustic measurements and BT (*r* = 0.99; *p* < 0.001) in melanocytic tumors including 6 melanomas [107], whereas the larger study by von Knorring et al. failed to reproduce the high accuracy reported previously, observing a tendency for PAI to overestimate melanoma thickness relative to histopathology [108].

Multispectral PAI acquires images at multiple wavelengths, enabling spectral unmixing of melanin and other chromophores. In the study by Park et al., melanin‐unmixed imaging demonstrated good agreement with histopathological BT, with a mean absolute error of 0.36 mm [109].

Ultra‐wideband Raster‐Scan Optoacoustic Mesoscopy (UWB‐RSOM) is a high‐resolution photoacoustic technique that combines optical contrast with mesoscopic imaging depth. Aguirre et al. reported an excellent correlation with BT (*R* ^2^ = 0.886; *p* = 0.0002), supporting its potential role in preoperative melanoma assessment [110].

### 3.6 MRI

MRI provides deep‐tissue evaluation of skin tumors, complementing superficial imaging modalities such as dermoscopy, RCM, and OCT. Advances in high‐resolution MRI, including dedicated surface coils and high‐field systems (1.5‐3 T), have improved the assessment of tumor thickness, margins, and local invasion [111].

The role of the MRI in BT estimation appears limited [112, 113].

In a retrospective study of 11 melanomas, Kang et al. evaluated high‐resolution MRI using a 1.5‐T scanner and microscopy coils [112]. T2‐weighted imaging (T2WI) showed better agreement with histopathological BT than gadolinium‐enhanced T1‐weighted imaging (Gd‐T1WI), with concordance correlation coefficients (CCC) ranging from 0.64 to 0.78 and intraclass correlation coefficients (ICC) from 0.93 to 0.95 [112]. In comparison, Gd‐T1WI demonstrated lower concordance (CCC 0.50‐0.61), despite similarly high ICC values (0.93–0.96) [112].

In an earlier case report, Nakayama‐Takeda et al. reported an almost exact correspondence between MRI‐measured tumor thickness (2.07 mm) and histopathological BT (2.06 mm) in a single melanoma [113].

### 3.7 SPECT

SPECT is a nuclear medicine imaging technique that generates three‐dimensional functional images by detecting gamma rays emitted from an injected radiotracer [114].

SPECT was evaluated in a single small prospective study using 99mTc‐sestamibi. Although its diagnostic performance for melanoma detection was modest (sensitivity 64.3% and specificity 66.7%), radiotracer uptake intensity showed a strong positive correlation with BT (rs = 0.74; *p* = 0.003), suggesting a potential role in estimating tumor depth [115].

### 3.8 AI/ML

AI and ML approaches applied to dermoscopic or multispectral images showed moderate to good performance for predicting melanoma invasiveness and stratifying BT, although results were heterogeneous across studies and tasks [33, 34, 35, 36, 37, 38, 39, 45, 46, 47, 103].

Most studies employed convolutional neural networks (CNNs), a class of deep learning algorithms specifically designed for image analysis [33, 34, 35]. CNNs automatically extract relevant image features from dermoscopic images and use them to classify melanomas according to BT categories. In the largest international study, Polesie et al. reported an overall accuracy of 56.4% for three‐class thickness classification, although accuracy was higher for thin (85.9%) and thick (70.8%) melanomas [33]. The collective performance of human readers (AUC 0.85) exceeded that of a de novo CNN (AUC 0.80) and was comparable to a pretrained CNN (AUC 0.83), suggesting that AI had not yet clearly surpassed expert assessment [33]. Similarly, Gillstedt et al. found that a CNN combining clinical and dermoscopic images achieved a lower AUC than experienced dermatologists (0.73 vs. 0.80; *p* = 0.001), with dermoscopic features such as shiny white lines, atypical blue‐white structures, and polymorphous vessels being strongly associated with invasive and thicker melanomas [34]. In contrast, Szijártó et al. demonstrated that an EfficientNet‐based CNN achieved an accuracy of 74.8% for three‐class BT prediction, outperforming previously reported ML models despite being trained on a relatively small dataset [35].

Additional AI approaches included transfer learning CNNs based on pretrained architectures such as VGG‐19 and EfficientNet [36, 39]. The VGG‐19 model developed by Jaworek‐Korjakowska et al. achieved an overall accuracy of 87.2% for three‐class BT prediction, with accuracies ranging from 85.5% to 89.3% across thickness categories [36]. Similarly, Hernández‐Rodríguez et al. reported accuracies of 61% for distinguishing melanoma in situ from invasive melanoma and 75% for predicting BT < 0.8 mm versus ≥0.8 mm using an EfficientNetB6 model [39].

Finally, artificial neural networks (ANNs) have also been investigated for BT prediction [37, 103]. Saez et al. developed the LIPU ANN model using dermoscopic features and reported an accuracy of 77.6% for binary thickness classification, outperforming logistic regression, support vector classifiers, kernel discriminant analysis, and alternative neural‐network architectures [37]. Performance decreased for multiclass classification, with an overall accuracy of 68.4% [37]. Using a different approach, Marchesini et al. applied ANNs to multispectral imaging data, achieving sensitivities ranging from 76% to 90% and specificities from 74% to 91% for distinguishing melanomas with BT below or above 0.75 mm [103].

Multispectral dermoscopy has also been explored as a noninvasive tool for estimating tumor depth. By analyzing lesion contrast across different wavelengths, early studies reported a significant correlation with histologic thickness, with the highest accuracy observed in the near‐infrared spectrum (940–950 nm), although a tendency to overestimate tumor depth was noted [45]. More recently, multispectral imaging features such as the presence of deep pigment have been associated with increased BT and showed good interobserver agreement (*κ* = 0.71), even though overall discriminatory performance remains limited [46]. Moreover, when combined with RCM, some specific features help in distinguish thin from thick lesions [47].

## 4 Discussion

This review evaluated the current evidence regarding non‐invasive imaging techniques for preoperative BT estimation in melanoma. Overall, the available literature suggests that while several modalities provide useful information on tumor depth, substantial differences exist in terms of technical performance, clinical maturity, accessibility, and level of validation.

Among the reviewed techniques, US demonstrated the strongest and most consistent evidence base, currently representing the most clinically mature modality for direct preoperative BT estimation, particularly when HFUS (20–40 MHz) or very‐high‐frequency US (VHFUS, ≥50 MHz) were used.

Appears to be the most promising emerging technology, whereas the rest of techniques evaluated primarily provide complementary information and currently play a more limited role in direct thickness assessment.

Dermoscopy remains the cornerstone of melanoma diagnosis because of its widespread availability, low cost, and high diagnostic accuracy. However, its ability to estimate BT is inherently indirect, relying on morphologic correlates of tumor invasion rather than direct measurement. Across studies, dermoscopic features such as blue‐white veil, shiny white structures, atypical vascular patterns, ulceration, and blue‐gray pigmentation consistently correlated with increasing thickness, reflecting biological progression from radial to vertical growth. Nevertheless, moderate interobserver agreement and reduced accuracy near clinically relevant thresholds limit its usefulness for surgical planning. Importantly, dermoscopy appears more effective in distinguishing clearly thick from thin lesions than in resolving subtle differences between melanoma in situ and early invasive melanoma. This limitation is clinically relevant because treatment decisions, including excision margins and SLNB indications, depend on distinctions occurring within the 0.8‐1.0 mm depth range.

RCM and OCT occupy an intermediate position between surface imaging and depth‐assessment techniques. Their major strength lies in their ability to provide near‐histologic visualization of tumor architecture and cellular morphology. The reviewed studies consistently demonstrated associations between increasing BT and features such as pagetoid spread, epidermal disarray, and non‐edged papillae. However, neither modality consistently achieved accurate direct thickness measurements. In particular, OCT was frequently limited by underestimation of tumor depth, likely because of its restricted penetration depth (0.5–2 mm) and signal attenuation in highly pigmented lesions. Consequently, RCM and OCT may be most valuable as adjunctive tools for lesion characterization and diagnostic refinement rather than as standalone methods for thickness estimation.

Compared with other imaging techniques, US demonstrated the most reliable and extensively validated performance for lesion thickness assessment, although its ability to characterize lesion nature remains modest. Multiple prospective and retrospective studies reported correlation coefficients exceeding 0.90 between sonographic and histopathologic thickness measurements, particularly when high‐frequency probes were used. In addition, US showed excellent performance for clinically relevant threshold‐based classification, often achieving sensitivities and specificities above 90% for distinguishing melanomas above and below 1 mm thickness. These findings are particularly important in view of future efforts to achieve single‐stage surgical management of melanoma [116]. Furthermore, US is relatively inexpensive, widely available, rapid, and easily integrated into routine clinical practice.

Nevertheless, several limitations should be acknowledged, including variability in performance across different US systems and acquisition settings. Although higher‐frequency probes generally provide greater spatial resolution, increasing probe frequency did not consistently translate into improved agreement with histopathology. Direct comparisons showed better performance with 20‐MHz than with lower‐frequency probes in some studies, whereas 20‐ and 50‐MHz systems yielded comparable correlations, and similarly high correlations were reported with 20‐ and 100‐MHz probes [60, 90, 98]. Thus, conventional US, HFUS, ultra‐high‐frequency US/VHFUS, and newer optically guided systems should not be considered directly interchangeable.

Operator‐dependent variability and standardization also remain relevant considerations. Although the limited available evidence on interobserver reproducibility is encouraging, most studies were conducted in single centers using different devices, probe frequencies, and acquisition and measurement protocols. In the 70‐MHz study, agreement across two operators was excellent (ICC = 0.99; CV 6.5%) [95], but this evidence remains limited compared with the much larger body of literature evaluating diagnostic accuracy itself. Consequently, the high diagnostic performance achieved in specialized settings cannot yet be assumed to be uniformly reproducible across different operators and ultrasound platforms.

Beyond these technical and operator‐related factors, intrinsic sources of measurement error also affect US accuracy. US frequently overestimated tumor thickness because of inflammatory infiltrates, fibrosis, regression, or adjacent nevus components [58, 60, 63, 68, 72, 74, 75, 77, 78, 79, 81, 82, 87, 92, 98], with accuracy being lower for melanoma in situ and very thin lesions using lower‐frequency (≤20 MHz) probes. Some studies also reported reduced reliability within the clinically relevant intermediate‐thickness range [77, 81]. These limitations further emphasize the need for standardized acquisition and measurement protocols, together with multicenter, cross‐device, interobserver, and real‐world validation, to establish the generalizability of US‐based BT estimation and identify the patient subgroups most likely to benefit from its use. Therefore, although HFUS currently represents the most extensively validated imaging approach for preoperative BT estimation, its performance remains influenced by technical, operator‐related, and lesion‐specific factors. Clinicians should therefore be aware of its tendency toward overestimation and reduced precision in selected melanoma subgroups, as well as the limited evidence regarding reproducibility across different devices and clinical settings.

MSI represents a conceptually attractive approach because it exploits wavelength‐dependent optical properties of melanin and hemoglobin without requiring tissue contact. The available studies reported moderate‐to‐good diagnostic performance, with sensitivities and specificities generally ranging between 75% and 90%. However, evidence remains limited to a small number of retrospective investigations. At present, MSI appears better suited as an adjunctive modality or as a source of quantitative features for dermoscopic or ML algorithms than as a standalone tool for thickness estimation.

Photoacoustic techniques are among the most promising developments identified in this review, since these systems theoretically may overcome some of the limitations of both conventional optical imaging (limited penetration) and US (in situ/thin melanomas’ BT misinterpretation due to inflammatory infiltrate). The ability to selectively visualize melanin‐rich structures may offer a unique advantage for melanoma assessment. However, the current evidence is derived largely from small, single‐center cohorts (Table 3), as well as the largest prospective study failed to replicate the exceptional accuracy reported previously. Therefore, larger multicenter studies are warranted to validate these findings before its implementation in routine clinical practice.

**TABLE 3:** Distribution of studies and sample sizes across non‐invasive imaging modalities for Breslow thickness estimation.

Technology	No. of studies	Total lesions ^a^	Melanomas
Dermoscopy	26	5.571	4070
AI/ML using dermoscopic images	7	5.623	5.623
US/HFUS	44	2.977	2.826
RCM/OCT	4	153	130
MSI	2	350	350
PAT/PAI	6	182	106
MRI	2	12	12
SPECT	1	20	14

^a^ Cumulative sample size excludes studies for which the exact number of evaluated lesions was not available.

Similarly, given the limited available evidence, high cost, technical complexity, and lack of extensive validation, MRI and SPECT do not currently appear suitable for routine preoperative assessment of tumor thickness.

AI represents an increasingly important area of investigation in dermatology. CNNs, transfer‐learning architectures, and ANN‐based approaches achieved moderate‐to‐good performance across multiple studies, with accuracies ranging from approximately 56% to 87%, predominantly using dermoscopic data. Notably, some transfer‐learning models achieved performance levels approaching those of expert clinicians. However, the largest comparative studies showed that experienced dermatologists matched or outperformed AI systems. Several factors may explain this performance gap. Current models are generally trained on relatively limited and heterogeneous datasets and predominantly analyze image‐derived information, whereas clinicians integrate dermoscopic morphology with additional contextual information, including clinical appearance, lesion history, anatomical site, patient characteristics, and pattern recognition acquired through experience. Moreover, differences in image acquisition, thickness thresholds, class imbalance, and limited external validation may reduce model generalizability, particularly around clinically critical thickness thresholds. Current AI models should therefore be regarded primarily as decision‐support tools rather than replacements for expert assessment. Future models integrating multimodal imaging and relevant clinical variables may provide a more clinically meaningful approach to preoperative thickness prediction.

From a practical perspective, the reviewed evidence supports a hierarchical rather than substitutive integration of non‐invasive imaging into the melanoma diagnostic workflow. Dermoscopy would remain the first‐line examination for lesion recognition and initial assessment of invasiveness, while RCM and OCT may provide additional microstructural information in selected diagnostically challenging lesions. When a melanoma is strongly suspected and preoperative thickness estimation could influence surgical planning, HFUS currently represents the most clinically applicable modality for direct assessment of tumor depth. Emerging techniques, such as PAI, may eventually complement this workflow by combining depth assessment with melanoma‐specific optical contrast, although their current level of validation does not support routine implementation. Such an approach could allow each modality to contribute according to its specific strengths rather than expecting a single technique to reproduce all the information provided by histopathology. In parallel, emerging molecular biomarkers, including circulating microRNAs, may in the future complement imaging‐based assessment and improve diagnostic and prognostic accuracy (e.g. miR‐146a‐5p, miR‐21‐5p) in melanoma [117, 118, 119, 120, 121].

Several limitations characterize the current evidence base. First, substantial heterogeneity exists across studies regarding thickness thresholds, outcome definitions, imaging protocols, and statistical methods. Second, many studies were retrospective, single‐center investigations with relatively small sample sizes, particularly for MRI, PAI, SPECT, and some RCM/OCT studies. Third, the lack of head‐to‐head studies precludes robust comparisons between imaging modalities and limits conclusions regarding their relative strengths and weaknesses. Fourth, histopathology itself is not free from variability, and tissue processing artifacts may influence comparisons with imaging‐derived measurements. Finally, the strength of the evidence should also be interpreted in relation to the markedly different sample sizes available for each modality (Table 3). While dermoscopy, AI‐based dermoscopic approaches, and US have been evaluated in cumulative cohorts comprising several thousand lesions, emerging technologies remain supported by substantially smaller populations. Therefore, high diagnostic performance reported in these preliminary studies should not be interpreted as equivalent in evidential strength to results reproduced across larger cohorts.

## 5 Conclusions

Accurate preoperative estimation of BT remains a clinically relevant yet challenging objective in melanoma management, with important implications for surgical planning, excision margins, and SLNB decisions.

Among the available modalities, US currently has the strongest evidence base and represents the most clinically applicable approach for direct thickness assessment. Dermoscopy, RCM, and OCT provide valuable information on melanoma invasiveness but are limited in their ability to accurately measure tumor depth, whereas MSI and PAI remain promising emerging technologies requiring further validation. AI has demonstrated encouraging results, particularly when integrated with dermoscopic or multispectral imaging, but current models do not consistently outperform expert clinicians.

Overall, a hierarchical approach is warranted, as no non‐invasive imaging modality can currently replace histopathological assessment. Nevertheless, advances in multimodal imaging and AI‐driven analysis may improve preoperative thickness estimation and support more personalized surgical management of melanoma.

## Funding

The article has the following funding source: Finanziato dall'Unione Europea NextGenerationEU a valere sul Piano Nazionale di Ripresa e Resilienza (PNRR)—Missione 4 Istruzione e ricerca—Componente 2 Dalla ricerca all'impresa—Investimento 1.1, Avviso Prin2022 indetto con DD N. 104 del 2/2/2022, codice proposta: 2022RY8549_002—CUP: J53D23003260006.

## Conflicts of Interest

The authors have no conflicts of interest to declare.

## Ethics Statement

Study did not require patient consent because no patients were involved.

## Supporting information




**Supporting Information**: srt70392‐sup‐0001‐SuppMat1.docx

## Data Availability

The data supporting the findings of this review are available within the article and its referenced sources. No new datasets were generated during the current study.
