# Supplementary material for: Non‐invasive Imaging Modalities for Breslow Thickness Estimation in Melanoma: A Scoping Review
Source: Skin Res Technol. 2026 Oct 14;32(10):e70392. doi: 10.1111/srt.70392 (PMC13662740; doi:10.1111/srt.70392)
Supplement: Supplementary file 1 [file SRT-32-e70392-s001.docx]

**Supplementary material S1**

**Pubmed**

“(Imaging OR non-invasive imaging OR dermoscopy OR videodermoscopy OR dermatoscopy OR super-high resolution dermoscopy OR super-high magnification dermoscopy OR 400x OR reflectance confocal microscopy OR RCM OR line-field confocal optical coherence tomography OR LC-OCT OR OCT OR high-frequency ultrasound OR HFUS OR ultra HFUS OR UHFUS OR high-resolution ultrasound OR high resolution ultra sound OR high resolution ultra-sound OR HRUS OR spectroscopy OR hyperspectral OR HIS OR multispectral OR electrical impedance spectroscopy OR EIS OR photoacoustic OR optoacoustic OR tomography OR PAT OR PAI OR mesoscopy OR RSOM OR thermographic OR terahertz pulsed OR TPI OR elastography OR multimodal) AND (Breslow OR thickness OR depth OR “tumor thickness") AND melanoma”

**Scopus**

“(Imaging [All Fields] OR “non-invasive imaging” [All Fields] OR dermoscopy [All Fields] OR videodermoscopy [All Fields] OR dermatoscopy [All Fields] OR “super-high resolution dermoscopy” [All Fields] OR “super-high magnification dermoscopy” [All Fields] OR 400x [All Fields] OR “reflectance confocal microscopy” [All Fields] OR RCM [All Fields] OR “line-field confocal optical coherence tomography” [All Fields] OR LC-OCT [All Fields] OR OCT [All Fields] OR “high-frequency ultrasound” [All Fields] OR HFUS [All Fields] OR ultra HFUS [All Fields] OR UHFUS [All Fields] OR “high-resolution ultrasound” [All Fields] OR “high resolution ultra sound” [All Fields] OR “high resolution ultra-sound” [All Fields] OR HRUS [All Fields] OR spectroscopy [All Fields] OR hyperspectral [All Fields] OR HIS [All Fields] OR multispectral [All Fields] OR “electrical impedance spectroscopy” [All Fields] OR EIS [All Fields] OR photoacoustic [All Fields] OR optoacoustic [All Fields] OR tomography [All Fields] OR PAT [All Fields] OR PAI [All Fields] OR mesoscopy [All Fields] OR RSOM [All Fields] OR thermographic [All Fields] OR “terahertz pulsed” [All Fields] OR TPI [All Fields] OR elastography [All Fields] OR multimodal [All Fields]) AND (Breslow [Article title, Abstract, Keywords] OR thickness [Article title, Abstract, Keywords] OR depth [Article title, Abstract, Keywords] OR “tumor thickness" [Article title, Abstract, Keywords]) AND (melanoma [Article title, Abstract, Keywords])”

**Web of Sciences**

“(TS=(imaging OR non-invasive imaging OR dermoscopy OR videodermoscopy OR dermatoscopy OR super-high resolution dermoscopy OR super-high magnification dermoscopy OR 400x OR reflectance confocal microscopy OR RCM OR line-field confocal optical coherence tomography OR LC-OCT OR OCT OR high-frequency ultrasound OR HFUS OR ultra HFUS OR UHFUS OR high-resolution ultrasound OR high resolution ultra sound OR high resolution ultra-sound OR HRUS OR spectroscopy OR hyperspectral OR HIS OR multispectral OR electrical impedance spectroscopy OR EIS OR photoacoustic OR optoacoustic OR tomography OR PAT OR PAI OR mesoscopy OR RSOM OR thermographic OR terahertz pulsed OR TPI OR elastography OR multimodal) AND TS=(Breslow OR thickness OR depth OR tumor thickness) AND TS=(melanoma))”
